# Normative reference values, determinants and regression equations for the incremental shuttle walk test (ISWT) in healthy Asian population aged 21 to 80 years

**DOI:** 10.1371/journal.pone.0291132

**Published:** 2023-09-05

**Authors:** Muhammad Zulhaziq Bin Azman, Katherin S. Huang, Wei Jun Koh, Sarah S. Leong, Benjamin Ong, Johanna L. Soon, Sherman W. Tan, Melissa Y. Chan, Mingxing Yang, Meredith T. Yeung

**Affiliations:** 1 Health and Social Sciences Cluster, Singapore Institute of Technology, Singapore, Singapore; 2 Department of Physiotherapy, Ng Teng Fong General Hospital, Singapore, Singapore; 3 Department of Physiotherapy, Sengkang General Hospital, Singapore, Singapore; 4 Department of Physiotherapy, Tan Tock Seng Hospital, Singapore, Singapore; 5 Department of Physiotherapy, Singhealth Polyclinics, Singapore, Singapore; Bangor University, UNITED KINGDOM

## Abstract

**Background:**

The validated Incremental Shuttle Walk Test (ISWT) is widely used for evaluating maximal exercise capacity, with the distance-walked (IWSD) as the primary outcome. However, there are no normative reference values (NRV) and reference equations to predict ISWD for the Singaporean population.

**Objectives:**

This study aims to establish the NRV and reference equations for ISWD in healthy Singaporeans aged 21 to 80 and investigate the determining variables during ISWT.

**Methods:**

This cross-sectional study recruited community-dwelling healthy subjects aged 21–80 from the community via convenience sampling. Each subject completed two trials of the ISWT according to the standard protocol. Variables measured during the trials included ISWD, pre-and post-test heart rate (HR), oxygen saturation, blood pressure (BP), modified Borg’s dyspnoea score and Borg’s rate of perceived exertion (RPE).

**Results:**

199 healthy Singaporean (females = 114, males = 85) participated in the study. The overall median ISWD was 660.0 metres (m) [interquartile range (IQR):440.0–850.0]. The age-stratified mean ISWD ranged from 430.0 m (IQR:350.0–450.0) (aged 60–80) to 480.0 m (IQR:438.0–650.0) (aged 40–59) to 780.0 m (IQR:670.0–960.0) (aged 21–39). Gender, age, weight, height and HR change (highest post-test HR minus pre-test HR) were the most significant variables (p < 0.001). IWSD (m) = 651.4(Height, m) +89.7(Gender, male = 1; female = 0) –6.31(Age, years) –3.61(Weight, kilograms) +2.54(HR change, beats per minute); R^2^ = 0.741. Previously published ISWT reference equations cannot accurately predict the ISWD in the Singaporean population.

**Conclusions:**

This study investigated the ISWD NRV and established reference equations for healthy Singaporeans aged 21–80. The information would be beneficial in setting performance benchmarks to guide physical assessment, intervention and rehabilitation.

## Introduction

The incremental shuttle walk test (ISWT) was developed by Singh et al. (1992) [1] as an alternative field test to evaluate the respiratory, cardiac and metabolic responses during exercise [2]. The externally-paced incremental walking test gauges maximal walking capacity and is inexpensive and straightforward to administer. It is a reliable and valid method of assessing the cardiorespiratory fitness of patients with chronic pulmonary disease (COPD) [1, 3, 4], cardiac disease [5, 6], peripheral arterial disease [7] and lung cancer survivors [8]. According to a systematic review by Singh et al. (2014), the ISWT was comparable to the cardiopulmonary exercise testing (CPET) in that both tests were progressive and had a strong correlation (r = 0.75–0.88) between the distance walked during the ISWT (ISWD) and maximal oxygen uptake (VO_2max_) [9]. Parreira et al. (2014) conducted another systematic review, demonstrating that the ISWT is a highly responsive test with good test-retest reliability of interclass correlation coefficient (ICC) of 0.75 to 0.99 in individuals with COPD and cardiovascular disease [10]. Some studies indicated a robust link between the ISWD and the distance walked in the 6-minute walk test by individuals with COPD; the correlation was strong, with ICCs ranging from 0.70 to 0.91 [4, 11–13]. The test has expanded its usage beyond just COPD patients. Nowadays, it is a standard outcome measure in clinical and research settings [14] for various medical conditions, including but not limited to obesity, bronchiectasis, cardiovascular disease, cancer and critical illness [10]. Normative reference values (NRV) for the ISWD, presented as distance-walked measured in metres, provide clinicians with comparative benchmark performance to healthy individuals.

Studies from various countries have produced NRV and derived the regression equations for healthy adults [15–19]. Several variables, including age, gender, height, weight, lung function, and maximal voluntary contraction of the quadriceps, were examined for their correlation with ISWD in these studies. All five studies found a significant correlation and variance (50.3–71%) between ISWD and age, gender, height, weight, and body mass index (BMI). However, only one of the studies analysed the physiological responses and the impact of age and gender on the cardiopulmonary load generated by the test [16]. The evaluation of the individual walking performance requires comparing it to the performance of a relevant population. This comparison necessitates the availability of normative reference values (NRV) specific to that population [20]. Different regression equations derived from different NRV would all result in different predictive ISWD, and it is uncertain if these values would over- or underestimate the predictive ISWD of any given local population. This idea was first suggested by Agarwal et al. (2016) [15], who found that the reported NRV of ISWD from the Indian population is vastly different when compared to the studies reported from Brazil [17] and the United Kingdom [16, 18] despite all existing normative reference ISWD values meant for the healthy population. Demographic and anthropometric profiles are common factors that influence ISWD [15, 17–19]. For example, ageing results in muscle loss and age-related degenerative changes that would reduce exercise capacity [21]. One report suggested that taller individuals tend to have a longer stride length [22], leading to a higher ISWD since stride length is a good indicator of walking speed. Conversely, individuals with a heavier body weight might have a shorter ISWD due to the increased workload they need to overcome while walking, which can result in decreased ISWD [17].

Hence, the current ISWT normative reference values and regression equations may not represent the Singaporean population due to differences in demographic and anthropometric profiles [23]. Therefore, this study aims to: (1) establish the NRV of ISWD in the healthy Singaporean population aged 21 to 80 years; (2) determine the correlations of variables that could influence the ISWD; (3) establish the ISWD regression equations applicable to healthy adult Singaporean; (4) evaluate the age-matched comparisons of the Singapore data with published studies.

## Methods

### Study design and recruitment process

Between July 2019 and March 2021, a convenience sampling cross-sectional study was conducted at various community centres in Singapore. Approval of the study was obtained from the University Institutional Review Board (Project number: 2019090). Written informed consent was obtained from each subject before participation; only anonymised data were used during data analysis. No access to the information that could identify subjects was necessary after data collection.

### Subjects

Healthy individuals aged 21 to 80 residing in various residential districts in Singapore were recruited consecutively. A sample size 97 was estimated with a confidence level of 95%, a 10% error margin and assumed 50% of the population proportion. We allowed for a possible attrition rate of 30%; thus, a minimum of 127 subjects were needed to distribute across the age range. After written consent, all subjects completed the baseline evaluation with the Physical Activity Readiness Questionnaire for Everyone (PAR-Q^+^) [24], vital signs measurements and pulmonary function tests before data collection. Inclusion criteria were: community ambulant adults between 21 to 80 years old as of testing day; able to understand simple English; BMI ≤ 27 kg/m^2^. Subjects were excluded if they had: inability to converse in simple English; any visual, auditory, or neuromuscular conditions such as amyotrophic lateral sclerosis, muscular dystrophy, myasthenia gravis; psychiatric or psychological disorders which could affect adherence to or comprehension of instructions; recent musculoskeletal injuries and/or surgery affecting gait and walking performance; the use of walking aids; any acute and/or chronic respiratory or cardiac disease or cancer in the last six months; resting heart rate (HR) > 100 beats per minute (bpm) or < 50 bpm; resting systolic blood pressure (SBP) > 150 or < 90 mmHg, diastolic blood pressure (DBP) > 100 or < 50 mmHg; oxygen saturation (SpO_2_) < 95% at rest on room air. Individuals with abnormal lung function, i.e., forced expiratory volume in 1 second (FEV_1_) ≤ 80%, forced vital capacity (FVC) ≤ 80%, and FEV_1_/FVC ≤ 70%, were also excluded.

### Data collection

Data collection was performed by researchers who were experienced in conducting the ISWT. The participants were instructed to wear comfortable, loose-fitting clothing and appropriate walking shoes. Data obtained from the subjects prior to the ISWT were: PAR-Q^+^, age, gender, height (Seca 213 portable stadiometer), weight (Omron digital weight scale, HN-286), spirometry [(FEV_1,_ FVC, FEV_1_/FVC), MIR Spirolab], resting BP (Welch Allyn, Gold Series DS66 Trigger Aneroids), smoking history, medical history and medication use. The calculation of BMI followed the standard formula [25]. HR & SpO_2_ (Nellcor^TM^, PM10N), dyspnea score using the modified Borg’s dyspnea scale [26], and Borg’s rating of perceived exertion (RPE) [27] were measured during the test as suggested by protocol [1]. Post-test BP was measured upon the immediate termination of the test.

### Incremental shuttle walk test

With adherence to the standardised ISWT instructions and protocol described by Singh et al. (1992) [1], the test was conducted within an open 10-metre course marked by two cones placed 0.5 metres (m) inwards from either end. The standard audio instructions were played before commencing the walking test. As the speed of walking increases every minute, indicated by a triple bleep, researchers advised the subjects, "You now need to increase your speed of walking". Subjects did not receive additional encouragement during the test unless they missed a shuttle; only then was a standardised prompt, "You need to increase your speed to keep up with the test", to encourage the subject to pick up their speed. The researchers demonstrated the test to the subjects before data collection, and there was no practice trial. Two trials of ISWT were conducted on the same day to account for the potential learning effect [9]. A minimum of 30 minutes was given between the two trials to allow for sufficient recovery [28] and to ensure that HR, BP and SpO_2_ returned to baseline levels before the second trial. The HR, BP, SpO_2_, Borg’s dyspnea scale, and RPE were recorded before and after each ISWT. During the test, HR and SpO_2_ were measured by a portable pulse oximeter (Nellcor^TM^ PM10N). The ISWT termination criteria for all age groups, as described in the standardised protocol [1], included: (1) HR exceeding maximal HR (HR_max_) using age-predicted HR_max_ = 220-age, (2) SpO_2_ falling below 80%, (3) inability to maintain the required speed due to dyspnea and/or leg fatigue, (4) missing two consecutive shuttles, and (5) when and if subjects indicate that they are unable to continue. The best distance walked was used for the analysis of NRV and the calculation of the regression equations.

### Statistical analysis

GraphPad Prism Version 8.4.3 (686) (GraphPad Software, San Diego, California, USA) was used for the statistical analysis. The level of significance was set at *p* < 0.05. Demographic and anthropometric data of subjects were examined for normal distribution using the Shapiro-Wilk test. Descriptive statistics were used to analyse the central tendency, data spread, and dataset position using the median, interquartile range (IQR), and 95% confidence interval (95%CI) of median. The Mann-Whitney U test was used to compare variables between genders, while the Kruskal-Wallis test was employed to analyse non-normally distributed continuous variables across different age groups. The interclass correlation coefficient (ICC) was used to evaluate test-retest reliability, while Pearson’s correlation coefficients were used to determine the correlation between variables. Linear regression was applied to establish the reference equations for ISWD from the data collected. In order to compare the measured ISWD from the current study with published reference equations, the age of participants was matched to the same age range of the calculated distance obtained from the predictive regression formulae of four identified reports. This allowed for comparing the age-matched Singapore data and the published reference equations [15, 17–19]. The measured and predicted ISWD comparisons were analysed using paired t-tests, and described with mean and standard deviation (SD).

## Results

### Subject characteristics and ISWD

Two hundred and fourteen subjects were recruited for initial assessment, and 15 were excluded based on exclusion criteria. Nil other subjects withdrew from the study. Therefore, 199 subjects (85 males; 114 females) were included for data analysis, observing a 6.95% error margin with a confidence level of 95% and assuming a 50% population proportion. Data imputation method was used for missing data during statistical analysis. Tables 1 and 2 present the subjects’ characteristics during ISWT stratified by gender and age groups. The overall median ISWD was 660.0 m [interquartile range (IQR) 440.0 to 850.0], where males walked 790.0 m (IQR 580.0–1020.0) compared to females 640.0 m (IQR 440.0–750.0) (p < 0.001) (Table 1). The IWSD decreased progressively with age and ranged from 780.0 m (IQR 670.0–960.0) (age 21–39) to 430.0 m (IQR 350.0–450.0) (age 60–80) (Table 2). Test-retest reliability was excellent (ICC = 0.91). The highest HR achieved was 157.0 bpm (IQR 135.0–177.0) from the 21–39 year group, while the %PredHRmax ranged from 80.0% (IQR 69.0–90.0) (age 21–39) to 84.0 (IQR 77.0–91.0) (age 60–80) (p = 0.281) (Table 2).

**Table 1:** Subjects’ characteristics during the ISWT (by gender).

Characteristics	Total	Male	Female	p-Value
*Subjects*, *n*	199	85	114	-
**Age (years)**	27.0 (IQR 23.0–61.0)	25.0 (IQR 24.0–54.0)	32.0 (IQR 23.0–62.0)	0.275
**Height (m)**	1.63 (IQR 1.59–1.69)	1.71 (IQR 1.66–1.76)	1.60 (IQR 1.53–1.64)	<0.001
**Weight (kg)**	59.6 (IQR 52.2–69.3)	66.5 (IQR 61.1–74.6)	54.8 (IQR 49.9–64.1)	<0.001
**BMI (kg/m**^**2**^**)**	22.2 (IQR 20.3–24.4)	23.0 (IQR 21.0–24.8)	21.8 (IQR 19.9–24.4)	= 0.007
**FEV**_**1**_**/FVC (%)**	82.9 (IQR 77.6–91.0)	86.5 (IQR 81.1–92.8)	80.9 (IQR 76.4–89.0)	= 0.002
***Vital sign measurements***				
**Resting HR (bpm)**	83.0 (IQR 74.0–91.0)	80.0 (IQR 70.5–87.0)	84.0 (IQR 75.0–93.0)	= 0.028
**Highest HR (bpm)**	142 (IQR 124.0–164.0)	146.0 (IQR 125.0–172.0)	142.0 (IQR 123.0–160.0)	= 0.425
**HRchange (bpm)**	62.0 (IQR 46.0–80.0)	69.0 (IQR 48.0–87.0)	57.5 (IQR 43.3–77.0)	= 0.074
**%PredHRmax**	82.0 (IQR 72.0–91.0)	80.0 (IQR 71.8–90.0)	83.0 (IQR 71.0–91.0)	= 0.557
***ISWD (m)***				
**ISWD1**	640.0 (IQR 430–780)	770.0 (IQR 560.0–920.0)	570.0 (IQR 370.0–673.0)	<0.001
95% CI	588.0 to 660.0	660.0 to 860.0	450.0 to 640.0	
**ISWD2**	660.0 (IQR 440–800)	780.0 (IQR 560.0–965)	580.0 (IQR 430.0–580.0)	<0.001
95% CI	590.0 to 670.0	720.0 to 890.0	460 to 650.0	
**ISWD2 –ISWD1**	20.0 (IQR -20-90)	10.0 (IQR -25.0–90.0)	20.0 (IQR -10.0–80.0)	0.959
95% CI	0.00 to 30.0	0.00 to 60.0	0.00 TO 40.0	
**Best of 2 trials**	660.0 (IQR 440.0–850.0)	790.0 (IQR 580.0–1020.0)	640.0 (IQR 440.0–750.0)	<0.001
95% CI	640.0 to 700.0	760.0 to 920.0	540 to 660.0	

Values are expressed as *median* and interquartile range (IQR), p < 0.05 represents a significant value; **m**: metres; **kg**: kilogram; **bpm**: beats per minute; **BMI**: Body mass index; **FEV1/FVC (%)**: Percentage of forced expiratory volume in the first one second to the forced vital capacity of the lungs; **ISWT**: Incremental shuttle walk test; **ISWD**: Incremental shuttle walk distance **HR**: Heart Rate; **Highest HR**: Highest heart rate achieved during ISWT; **HRchange**: Difference between Highest HR heart rate and resting heart rate; **%PredHRmax**: peak HR achieved during ISWT expressed as %predicted maximum HR with predicted HRmax as (220-age); **95% CI**: 95% Confidence Interval; **ISWD1**: 1^st^ trial of ISWD; **ISWD2**: 2^nd^ trial of ISWD.

**Table 2:** Subjects’ characteristics during the ISWT (by age).

Characteristics	Age			p-Value
	21–39 years	40–59 years	60–80 years	
***Subjects*,** *n*	122	26	51	
**Height (m)**	1.67 (IQR 1.61–1.73)	1.61 (IQR 1.55–1.66)	1.59 (IQR 1.52–1.63)	<0.001
**Weight (kg)**	62.5 (IQR 54.3–71.8)	57.9 (IQR 52.9–65.6)	54.5 (IQR 49.6–64.1)	= 0.002
**BMI (kg/m2)**	22.1 (IQR 20.2–24.0)	22.9 (IQR 20.7–25.0)	22.2 (IQR 20.1–24.7)	= 0.622
**FEV1/FVC (%)**	89.0 (IQR 81.1–94.1)	81.1 (IQR 76.7–84.1)	77.3 (IQR 73.4–81.8)	<0.001
***Vital sign measurements***				
**Resting HR (bpm)**	86.0 (IQR 77.0–93.0)	76.0 (IQR 68.0–93.5)	80.0 (IQR 70.0–87.0)	= 0.032
**Highest HR (bpm)**	157.0 (IQR 135.0–177.0)	142.0 (IQR 118.0–156.0)	129.0 (IQR 115.0–141.0)	<0.001
**HRchange (bpm)**	76.0 (IQR 57.0–89.0)	50.0 (IQR 46.5–71.5)	49.0 (IQR 33.0–60.0)	<0.001
**%PredHRmax**	80.0 (IQR 69.0–90.0)	83.0 (IQR 71.0–91.0)	84.0 (IQR 77.0–91.0)	0.281
***ISWD (m)***				
**ISWD1**	715.0 (IQR 650–900)	455.0 (IQR 363.0–618.0)	360.0 (IQR 320.0–440.0)	<0.001
95% CI	680.0 to 780.0	370 to 580.0	350.0 to 380.0	
**ISWD2**	770.0 (IQR 660.0–928.0)	455.0 (IQR 410.0–558.0)	400.0 (IQR 340.0–450)	<0.001
95% CI	730.0 to 800.0	440.0 to 550.0	350.0 to 430.0	
**ISWD2 –ISWD1**	20.0 (IQR -12.5–100)	0.00 (IQR -82.5–82.5)	10.0 (IQR -10.0–70.0)	= 0.295
95% CI	0.00 to 40.0	-70.0 to 70.0	0.00 to 50.0	
**Best of 2 trials**	780.0 (IQR 670.0–960.0)	480.0 (IQR 438.0–650.0)	430.0 (IQR 350.0–450.0)	<0.001
95% CI	760.0 to 840.0	440.0 to 640.0	360.0 to 440.0	

Values are expressed as *median* and interquartile range (IQR), p < 0.05 represents a significant value; **m**: metres; **kg**: kilogram; **bpm**: beats per minute; **BMI**: Body mass index; **FEV1/FVC (%)**: Percentage of forced expiratory volume in the first one second to the forced vital capacity of the lungs; **ISWT**: Incremental shuttle walk test; **ISWD**: Incremental shuttle walk distance; **HR**: Heart Rate; **Highest HR**: Highest heart rate achieved during ISWT; **HRchange**: Difference between Highest HR and resting heart rate; **%PredHRmax**: peak HR achieved during ISWT expressed as %predicted maximum HR with predicted HRmax as (220-age); **95% CI**: 95% Confidence Interval; **ISWD1**: 1^st^ trial of ISWD; **ISWD2**: 2^nd^ trial of ISWD.

### Relationship between ISWD and variables

The correlations between ISWD, demographics and anthropometric variables are depicted in Table 3. Only BMI, resting HR and %PredHRmax were not correlated to ISWD. Age, gender, height, weight and FEV_1_/FVC are standard parameters assessed before and during the ISWT assessment, as protocol suggests [1]. This suggests the feasibility and usefulness of estimating and benchmarking the outcome measures. Post-test variables, namely Highest HR and HR change (difference between highest HR measured during the test–resting HR), attained statistically significant (p < 0.001).

**Table 3:** Univariate correlation coefficients (r) for ISWD and subject variable(s) (n = 199).

Variable	r	95% CI	p-Value
**Age (years)**	-0.62	-0.7047 to -0.5281	<0.001*
**Gender**	0.39	0.2595 to 0.5040	<0.001*
**Height (m)**	0.58	0.4718 to 0.6648	<0.001*
**Weight (kg)**	-0.27	-0.1322 to -0.3986	<0.001*
**BMI (kg/m** ^**2**^ **)**	-0.05	-0.1889 to 0.0974	= 0.51
**FEV**_**1**_ **/FVC (%)**	0.51	0.3836 to 0.6221	<0.001*
**Resting HR (bpm)**	0.06	-0.0877 to 0.2242	= 0.37
**Highest HR (bpm)**	0.58	0.4651 to 0.6750	<0.001*
**HR change (bpm)**	0.62	0.5117 to 0.7070	<0.001*
**%PredHRmax**	0.14	-0.0209 to 0.2869	= 0.08

*p < 0.05 represents a significant value; **95% CI**: 95% Confidence

**m**: metres; **kg**: kilogram; Body mass index; **FEV1/FVC (%)**: Percentage of forced expiratory volume in the first one second to the forced vital capacity of the lungs; **bpm**: beats per minute; **ISWD**: Incremental shuttle walk distance; **HR**: Heart Rate; **%PredHRmax**: the percentage that highest HR achieved out of HRmax [HRmax derived from 220-age]

### Regression equations between ISWD and variables

Multiple linear regression analysis revealed that among the pre-test variables, gender, age, weight, and height alone explained 67.2% of the variance (Table 4). However, with the addition of the post-test variable (HR change), the percentage of the variance increased to 74.1% (Table 5).

**Table 4:** Linear regression model and equation for predicting ISWD with pre-test variables.

ISWD = 790.9 (Height, m) + 86.1 (Gender, male = 1; female = 0)– 7.43 (Age, years)– 3.70 (Weight, kg); R^2^ = 0.672				
R^2^ = 0.672	Coefficient	Standard error	P-value	95% CI
**Constant**	-224.4	373.2	= 0.549	-961.7 to 513.0
**Gender**	86.1	32.82	= 0.009*	21.3 to 150.9
**Age**	-7.43	0.768	< 0.001*	-8.95 to -5.91
**Weight**	-3.70	1.184	= 0.002*	-6.04 to -1.36
**Height**	790.9	212.6	< 0.001*	370.8 to 1121.0
**FEV**_**1**_ **/FVC(%)**	0.973	1.475	= 0.510	-1.94 to 3.89

*p < 0.05 represents a significant value; **ISWD**: Incremental shuttle walk distance; **95% CI**: 95% Confidence Interval; **FEV1/FVC (%)**: Percentage of forced expiratory volume in the first one second to the forced vital capacity of the lungs; Gender: (1 = male, 0 = female).

**Table 5:** Linear regression model and equation for predicting ISWD with pre-and post-test variables.

ISWD = 651.4 (Height, m) + 89.7 (Gender, male = 1; female = 0)– 6.31 (Age, years)– 3.61 (Weight, kg) + 2.54 (HR change, bpm); R^2^ = 0.741				
R^2^ = 0.741	Coefficient	Standard error	P-value	95% CI
**Constant**	-184.3	346.0	= 0.595	-867.9 to 499.4
**Gender**	89.7	29.64	= 0.003*	31.1 to 148.3
**Age**	-6.31	0.721	< 0.001*	-7.73 to -4.88
**Weight**	-3.61	1.059	< 0.001*	-5.70 to -1.52
**Height**	651.4	191.5	< 0.001*	272.9 to 1030.0
**FEV**_**1**_ **/FVC(%)**	0.10	1.33	= 0.938	-2.52 to 2.72
**HR change**	2.54	0.943	= 0.008*	0.68 to 4.40

*p < 0.05 represents a significant value; **ISWD**: Incremental shuttle walk distance; **95% CI**: 95% Confidence Interval; **FEV1/FVC (%):** Percentage of forced expiratory volume in the first one second to the forced vital capacity of the lungs; Gender: (1 = male, 0 = female); **HR**: Heart Rate; **HRchange**: Difference between HighestHR heart rate and resting heart rate.

### Comparisons with the ISWD estimated using the previously published equations

Table 6 illustrates the age-matched measured data from this study compared to the age-matched ISWD calculated using the predictive formulae from 4 previous similar studies [15, 17–19]. The distance walked by the subjects in this study was shorter in three out of the four studies, even though only one attained statistical significance (p < 0.001). On the contrary, the distance walked in this study was underestimated by 157.0 ± 152.0 m with the regression formula established by Agarwal et al. (2016) [15].

**Table 6:** Age-matched comparison between measured ISWD from this study and predicted ISWD from reference equations of published studies.

Study	Country	Measured ISWD (m) Mean ± SD	Predicted ISWD (m) Mean ± SD	Difference (m) (Measured–Predicted) Mean ± SD	P-value
Jurgensen et al. (2011)^18^, 40–84 years ISWD (m) = 374.0–6.782(age, year)– 2.328 (weight, kg) + 3.865 (height, m) + 115.937 (gender: male = 1; female = 0)	Brazil	467.0 ± 145.0	484.0 ± 87.0	-17.3 ± 132.0	= 0.255
Probst et al. (2012)^19^, 18–83 years ISWD (m) = 1449.701–11.735 (age, year) + 241.897 (gender: male = 1, female = 0)	Brazil	666.0 ± 233.0	955.0 ± 259.0	-289.0 ± 157.0	<0.001*
Agarwal et al. (2016)^15^, 17–75 years ISWD (m) = 740.351–5.676 (age, year) + 99.007 (gender: male = 1; female = 0)	India	800.0 ± 179.0	643.0 ± 57.0	157.0 ± 152.0	<0.001*
Itaki et al. (2018)^17^, 20–90 years ISWD (m) = -4.894–4.107 (Age, years) + 131.115 (gender: male = 1; female = 0) + 4.895 (height, centimetres)	Japan	666.0 ± 233.0	689.0 ± 140.0	-22.8 ± 155.0	= 0.162

Values expressed as *mean* ± *Standard Deviation (SD)*

*p<0.05 represents a significant difference between age-matched subjects measured vs predicted ISWD. **ISWD**: Incremental shuttle walk distance, m: metres; kg: kilograms

## Discussion

This study produced the NRV and developed regression equations for ISWD of healthy Singaporeans aged 21 to 80. This study reported the overall mean ISWD, with male subjects walking significantly longer distances than female subjects. Furthermore, the mean ISWD decreased progressively with the advancement of age. The high ICC for repeated tests demonstrated good test-retest reliability. The ISWT is influenced by the learning effect [9, 29] as the second test distance was consistently higher than the first test by no more than 6%, except for group age 40–59 [0.00 m (IQR -82.5–82.5) 95% CI -70 to 70]. To mitigate the influence of the learning effect, this study employed a procedure where two standardised tests were conducted on the same day. The better result between the two tests was then selected for analysis to avoid potential biases.

Several factors significantly influenced the ISWD in this study, including age, gender, height, weight, FEV_1_/FVC, Highest HR, HR change, and %PredHRmax (Table 3). On the other hand, BMI and resting HR were not statistically significant predictors of ISWD. The impact of age, gender, height, and weight on ISWD has been extensively reported in previous studies concerning reference equations for ISWD [15–19]. The negative correlation between age and ISWD observed in this study may be attributed to a decline in physical fitness associated with ageing. Previous research has consistently shown that ageing is associated with muscle loss, reduction in maximum oxygen consumption (VO_2max_), decreased stride length, and alterations in gait. These age-related changes in musculoskeletal and cardiovascular function can collectively contribute to a decline in ISWD performance [21, 30, 31]. Ageing leads to muscle loss and a decrease in maximum oxygen consumption (VO_2max_) [21], which can affect overall physical fitness and performance in activities such as the ISWD. Similarly, it is well-established that females generally exhibit lower cardiovascular and muscular fitness levels than males [15–19].

In this study, the age group 21–39 recorded the highest ISWD of 780.0 m (IQR 670.0–960.0) despite the highest average height [1.67 m (IQR 1.61–1.73)] and weight [62.5 kg (IQR 54.3–71.8)] across the age groups. Jurgensen et al. (2001) previously proposed that taller individuals are likelier to have a longer stride length. This longer stride length enables them to walk faster, potentially resulting in a longer ISWD [17]. Noteworthily, weight was found to have a negative correlation (r = -0.27; p < 0.001) with ISWD. However, the magnitude of this influence might be compensated for by the influences of age (r = -0.62, p < 0.001) and height (r = 0.58, p < 0.001). In other words, although weight was negatively associated with ISWD, the effects of age and height on ISWD were more substantial and could potentially offset the negative impact of weight. These factors can collectively contribute to this study’s observed negative association between age, weight and ISWD [15–19]. Hence, a congruent trend observed in the current data reveals that male subjects walked an average of 790 m (IQR 580.0–1020.0) vs 640.0 m (IQR 440.0–750.0), with the female subjects [Table 1 (male: height = 1.71 m & weight = 66.5 kg; female: height = 1.60 m & weight = 54.8 kg, p < 0.001)].

Our subjects achieved 82.0% (IQR 72.0–91.0) of predicted maximal heart rate (%HRmaxPred) during the ISWT (Table 1), derived from the formula: *highest HR achieved during ISWD* ÷ *(220-Age)*, indicating vigorous-intensity effort, consistent with previous studies [15–19] and the maximum nature of this field test. This value is comparable to previous reports of 78% to 81% [17, 19], which used the standard ISWT protocol [1, 17, 19], but considerably lower than the 99% reported by Probst et al. (2012), who used the modified version of the ISWT protocol instead [18]. The modified protocol extended the ISWT over three additional levels by increasing the speed by 0.17 metres per second (m/s) each minute [32], thus explaining the much higher %HR_max_Pred.

Similar to the previous studies, we reported that age, gender, weight and height were statistically significant variables that estimate the ISWD. These simple pre-test variables alone can explain a significant amount of variance, up to 67.2%, indicating their predictive value and practical applicability in interpreting ISWD results. These variables provide specific information for understanding ISWT outcomes. Additionally, this equation holds particular relevance for individuals who are taking chronotropic agents, as these medications have the potential to influence the regularity and rhythm of heart rate, which in turn may impact the extent of heart rate variation during the ISWT. In contrast, the second reference equation comprised the addition of HR change and established a higher percentage of variance (R^2^ = 74.1%), which would be helpful for assessors to benchmark the ISWT performance for other individuals.

Our ISWD reference equation explained 67.2% to 74.1% of the variance, similar to findings from other studies, ranging from 50% to 78.2% [15, 17–19], despite the different age groups of the subjects. Four published studies [15, 17–19] were selected to perform an age-matched comparison. These studies were utilised to compare the measured ISWD obtained from the current study with the predicted ISWD values derived from the regression equations mentioned. By employing this approach, a comprehensive assessment of how well the measured ISWD aligns with the predicted values can be made, providing valuable insights into the accuracy and reliability of those regression equations to our local population. Another formula derived by Harrison et al. (2013) contained additional variables unavailable in this current study and was therefore omitted from the comparison. Of the four studies being compared, the formulae from Probst et al. (2012) and Agarwal et al. (2016) were found respectively over-and under-estimate the ISWD of our subjects significantly (-289.0 ± 157.0 m, p < 0.001; 157.0 ± 152.0, p < 0.001). Furthermore, the variables in our equation, namely age, gender, height and weight, are readily available before the commencement of the test, thus highlighting the potential predictive application of the formula.

Some limitations of this study should be considered. Firstly, this study recruited 114 females of the 199 subjects (57.3%) and 122 subjects from the 21–39 age group (61.3%). The age groups 40–59 and 60–80 combined contributed to the remaining 38.7% of the overall sample size. This possibly skewed the results to the 21–39 age group and female subjects with an over-representation. There was also a 25.9% variance that the existing data could not explain. Future studies should aim to identify and incorporate additional variables that may contribute to the unexplained variance in ISWD. By considering these missing variables, researchers can potentially improve the predictive models and provide a more comprehensive understanding of the factors influencing ISWD. This could lead to more accurate assessments and better interpretations of ISWD outcomes in clinical and research settings.

## Conclusions

This study represents a pioneering effort to establish normative reference values and regression equations for the Incremental Shuttle Walk Test in healthy Singaporean adults aged 21 to 80. The results revealed significant associations between ISWD and age, gender, height, weight, and heart rate change. It is crucial to note that utilising equations derived from other studies may lead to inaccurate estimations of ISWD in the Singaporean population, given the presence of population-specific variations. Therefore, this study’s value and reference equation hold substantial merit in establishing performance benchmarks and guiding interventions and rehabilitation strategies. Future follow-up studies should investigate the factors contributing to the unexplained variance observed in the developed equations, thus enhancing our understanding of the determinants of ISWD in this population.

## Supporting information

S1 Table Demographics and variables measured from subjects participated in ISWT. (PDF)
