# Supplementary material for: Normative reference values, determinants and regression equations for the incremental shuttle walk test (ISWT) in healthy Asian population aged 21 to 80 years
Source: PLoS One. 2023 Sep 5;18(9):e0291132. doi: 10.1371/journal.pone.0291132 (PMC10479918; doi:10.1371/journal.pone.0291132)
Supplement: S1 Table — (PDF) [file pone.0291132.s001.pdf]

Table format: **XY**		X<br>Total distance	Group A<br>Gender_coded	Group B<br>Age	Group C<br>Weight	Group D<br>Height	Group E<br>BMI	Group F<br>FEV1/FVC (%)	Group G<br>HR change
		X	Y	Y	Y	Y	Y	Y	Y
1	Title	1020	1	21	77.30	1.76	25.00	90.0	93
2	Title	760	0	22	53.30	1.64	19.94		
3	Title	680	0	22	51.00	1.58	20.43		
4	Title	860	0	22	53.00	1.55	22.10	91.0	98
5	Title	860	0	22	53.00	1.55	22.10	91.0	89
6	Title	900	0	22	57.10	1.73	19.10	84.2	66
7	Title	780	0	22	49.50	1.65	18.30	106.0	93
8	Title	770	0	22	78.20	1.65	28.70	89.0	99
9	Title	640	0	22	45.50	1.58	18.34	79.2	89
10	Title	780	0	22	56.30	1.63	21.20	98.0	98
11	Title	560	0	22	47.30	1.60	18.45	94.4	80
12	Title	780	0	22	49.90	1.53	21.21		16
13	Title	680	0	22	44.10	1.50	19.65	89.6	77
14	Title	800	0	22	47.80	1.59	19.03	87.9	17
15	Title	690	0	22	59.60	1.63	22.51	78.7	77
16	Title	450	0	22	44.10	1.52	19.09	80.5	25
17	Title	640	0	22	50.00	1.64	18.54	79.3	65
18	Title	450	0	22	67.10	1.64	25.10	99.0	86
19	Title	660	0	22	66.10	1.64	24.46	67.8	32
20	Title	770	0	22	61.40	1.63	23.10	100.0	90
21	Title	960	0	22	45.20	1.58	18.10	91.0	99
22	Title	790	0	22	66.00	1.68	23.40	91.0	82
23	Title	660	0	22	39.10	1.50	17.40	100.0	82
24	Title	670	0	22	55.40	1.57	22.53	83.8	68
25	Title	670	1	22	64.80	1.71	22.20	96.0	53
26	Title	940	1	22	72.90	1.75	23.70	99.0	86
27	Title	880	1	22	67.70	1.74	22.34	81.1	91
28	Title	970	1	22	81.30	1.83	24.22	77.6	96
29	Title	650	0	23	77.40	1.69	27.07		
30	Title	660	0	23	50.40	1.60	19.74		
31	Title	690	0	23	56.20	1.72	19.11		
32	Title	660	0	23	46.00	1.67	16.57		
33	Title	620	0	23	51.80	1.61	20.06		
34	Title	800	0	23	64.20	1.67	22.99	76.7	57
35	Title	430	0	23	70.00	1.58	28.00	77.1	51
36	Title	770	0	23	48.80	1.65	17.90	105.0	106
37	Title	680	0	23	52.20	1.64	19.48	86.4	52
38	Title	920	0	23	68.10	1.64	25.29	82.3	81
39	Title	910	0	23	54.60	1.61	21.10	94.0	76
40	Title	640	0	23	62.40	1.62	23.92	80.8	29
41	Title	660	0	23	51.50	1.60	20.09	100.0	59
42	Title	720	1	23	63.20	1.69	22.13		

Table format: XY		X	Group A	Group B	Group C	Group D	Group E	Group F	Group G
		Total distance	Gender_coded	Age	Weight	Height	BMI	FEV1/FVC (%)	HR change
		X	Y	Y	Y	Y	Y	Y	Y
43	Title	770	1	23	57.50	1.65	21.04		
44	Title	1020	1	23	68.90	1.74	22.89		
45	Title	670	1	23	72.90	1.76	23.67		
46	Title	650	1	23	62.60	1.71	21.53		
47	Title	1020	1	23	66.50	1.72	22.50	98.0	109
48	Title	1020	1	23	71.80	1.77	22.97	87.0	71
49	Title	960	1	23	62.50	1.75	20.45		84
50	Title	710	1	23	80.00	1.70	27.70	97.0	76
51	Title	840	1	23	71.10	1.68	25.20	94.0	117
52	Title	770	1	23	62.20	1.71	21.30	95.0	60
53	Title	920	1	23	63.80	1.81	19.56	100.0	64
54	Title	1020	1	23	89.40	1.74	29.50	83.0	87
55	Title	850	1	23	69.00	1.71	23.74	88.1	18
56	Title	1020	1	23	66.20	1.83	19.70	99.0	21
57	Title	790	0	24	53.90	1.63	20.30	100.0	94
58	Title	750	0	24	57.80	1.62	22.00	80.3	101
59	Title	640	0	24	42.80	1.54	18.00	91.0	43
60	Title	770	0	24	55.30	1.61	21.47	79.9	73
61	Title	580	1	24	86.50	1.77	27.49		
62	Title	660	1	24	92.80	1.69	32.65		
63	Title	730	1	24	66.00	1.73	22.05		
64	Title	780	1	24	78.80	1.68	27.79		
65	Title	1020	1	24	59.70	1.69	20.93		
66	Title	1020	1	24	76.20	1.78	24.00	86.0	70
67	Title	890	1	24	85.00	1.77	27.10	95.0	75
68	Title	1020	1	24	57.90	1.68	20.64	93.6	78
69	Title	770	1	24	52.70	1.62	20.21		77
70	Title	1020	1	24	70.60	1.83	21.10	87.0	63
71	Title	760	1	24	86.80	1.74	28.70	92.0	52
72	Title	810	1	24	64.70	1.64	24.03	84.0	45
73	Title	1020	1	24	75.90	1.71	26.05	89.3	111
74	Title	910	1	24	107.20	1.85	31.25		46
75	Title	1020	1	24	85.00	1.86	24.60	91.0	87
76	Title	440	0	25	88.60	1.60	34.83	76.3	39
77	Title	690	0	25	49.00	1.52	21.24	97.5	13
78	Title	460	0	25	56.30	1.61	21.77	70.1	53
79	Title	780	1	25	66.70	1.67	23.80		
80	Title	1020	1	25	89.30	1.78	28.25		
81	Title	910	1	25	59.10	1.73	19.84		
82	Title	780	1	25	75.50	1.81	22.94		
83	Title	920	1	25	62.80	1.74	20.70	89.0	88
84	Title	750	1	25	63.00	1.63	23.71	95.7	52

Table format: XY		X	Group A	Group B	Group C	Group D	Group E	Group F	Group G
		Total distance	Gender_coded	Age	Weight	Height	BMI	FEV1/FVC (%)	HR change
		X	Y	Y	Y	Y	Y	Y	Y
85	Title	790	1	25	66.00	1.69	23.10	93.0	65
86	Title	1020	1	25	71.90	1.77	22.87	89.5	95
87	Title	1020	1	25	70.30	1.75	22.96	93.0	87
88	Title	1020	1	25	73.30	1.83	21.90	83.0	69
89	Title	980	1	25	63.80	1.64	23.72	86.9	88
90	Title	920	1	25	85.20	1.67	30.70	88.0	69
91	Title	660	0	26	58.30	1.61	22.50	107.0	58
92	Title	670	0	26	60.90	1.63	22.90	89.0	-16
93	Title	1020	1	26	54.40	1.66	19.81		
94	Title	1020	1	26	71.70	1.71	24.52		
95	Title	780	1	26	60.20	1.81	18.29	72.4	73
96	Title	990	1	26	79.00	1.73	26.40	91.0	105
97	Title	640	1	26	62.40	1.70	21.69	90.2	49
98	Title	580	0	27	56.10	1.60	22.00		
99	Title	700	0	27	50.10	1.57	20.35		
100	Title	360	0	27	108.30	1.67	38.83	71.0	33
101	Title	1020	0	27	55.60	1.68	19.77	80.8	94
102	Title	570	1	27	51.30	1.61	19.86		
103	Title	1020	1	27	79.90	1.70	27.52	85.2	62
104	Title	1020	1	27	58.30	1.75	19.06	91.5	61
105	Title	610	0	28	50.70	1.62	19.32		
106	Title	1020	1	29	55.00	1.63	20.75	82.9	70
107	Title	910	0	30	49.60	1.53	21.33	88.8	65
108	Title	1020	1	30	73.40	1.77	23.40	80.2	100
109	Title	1020	0	31	66.10	1.69	23.12		
110	Title	910	0	31	56.80	1.68	20.12	79.9	62
111	Title	910	0	31	57.60	1.60	22.58	81.0	94
112	Title	890	1	31	58.40	1.63	22.06		
113	Title	810	0	32	49.10	1.60	19.20	100.0	76
114	Title	680	0	32	56.90	1.61	22.01		97
115	Title	1020	1	32	73.70	1.79	22.98		
116	Title	1020	1	32	59.40	1.68	20.95	86.0	90
117	Title	670	0	33	59.20	1.63	22.23	87.4	89
118	Title	790	1	33	70.30	1.66	25.64		
119	Title	660	0	34	50.70	1.57	20.49	79.9	69
120	Title	370	0	35	73.00	1.69	25.44	93.6	35
121	Title	560	0	36	82.50	1.67	29.65	70.5	78
122	Title	710	0	39	65.10	1.65	23.91	84.1	84
123	Title	440	0	40	82.10	1.59	32.43	76.3	64
124	Title	650	0	41	52.80	1.65	19.30	85.5	41
125	Title	680	0	42	65.10	1.55	27.24		
126	Title	1020	1	42	57.90	1.67	20.69	95.1	93

Table format: **XY**		X	Group A	Group B	Group C	Group D	Group E	Group F	Group G
		Total distance	Gender_coded	Age	Weight	Height	BMI	FEV1/FVC (%)	HR change
		X	Y	Y	Y	Y	Y	Y	Y
127	Title	440	0	44	54.10	1.60	21.13	84.0	50
128	Title	340	0	46	64.80	1.61	25.00	71.0	51
129	Title	430	0	47	54.80	1.54	23.02	78.4	46
130	Title	640	0	48	57.90	1.63	21.79	83.7	48
131	Title	580	0	48	64.00	1.68	22.78	75.9	44
132	Title	430	1	48	77.80	1.68	27.53	81.1	70
133	Title	370	0	53	52.90	1.45	25.06	78.9	20
134	Title	770	0	53	54.80	1.61	21.17	83.3	81
135	Title	530	1	54	61.30	1.60	24.01	84.1	72
136	Title	470	1	54	65.00	1.64	24.20	97.4	28
137	Title	440	0	55	40.20	1.53	17.15	86.2	47
138	Title	440	0	55	52.10	1.52	22.70	77.0	48
139	Title	460	0	55	67.00	1.65	24.73	84.9	57
140	Title	530	0	55	48.20	1.53	20.54	71.2	91
141	Title	270	0	56	79.00	1.64	29.34	82.5	47
142	Title	260	1	56	63.20	1.72	21.36	81.1	7
143	Title	460	0	57	69.30	1.59	27.27	66.0	50
144	Title	760	1	57	55.80	1.68	19.89	81.6	76
145	Title	610	0	58	50.90	1.45	24.21	77.7	67
146	Title	650	0	59	54.20	1.66	19.67	74.6	74
147	Title	490	1	59	47.30	1.61	18.27	78.7	47
148	Title	760	1	59	72.50	1.72	24.39	79.6	71
149	Title	540	0	60	51.50	1.63	19.38	74.9	20
150	Title	350	0	61	49.40	1.50	21.90	78.6	50
151	Title	450	0	61	67.40	1.59	26.79	80.0	68
152	Title	430	0	61	54.90	1.62	20.94	67.6	63
153	Title	580	1	61	62.80	1.67	22.52	72.4	68
154	Title	430	0	62	59.80	1.63	22.45	73.9	34
155	Title	370	0	62	53.30	1.60	20.72	77.8	21
156	Title	590	0	63	47.20	1.63	17.77	80.4	60
157	Title	440	0	63	53.90	1.63	20.29	67.9	54
158	Title	250	1	63	78.70	1.77	25.15	81.3	89
159	Title	430	0	64	46.90	1.53	19.96	100.0	59
160	Title	740	1	64	78.10	1.82	23.47	74.0	77
161	Title	400	0	65	51.60	1.55	21.37	76.8	50
162	Title	440	0	65	54.70	1.50	24.44	76.8	48
163	Title	280	0	65	69.40	1.44	33.38	81.3	31
164	Title	370	0	66	66.40	1.51	29.12	73.4	57
165	Title	370	0	66	49.10	1.50	21.76	83.4	45
166	Title	210	0	66	46.20	1.52	19.94	75.3	30
167	Title	350	0	66	64.10	1.52	27.74	78.3	27
168	Title	440	0	66	44.60	1.53	19.08	82.2	35

Table format: XY		X	Group A	Group B	Group C	Group D	Group E	Group F	Group G
		Total distance	Gender_coded	Age	Weight	Height	BMI	FEV1/FVC (%)	HR change
		X	Y	Y	Y	Y	Y	Y	Y
169	Title	350	1	66	71.20	1.63	26.80	87.2	40
170	Title	440	0	68	54.70	1.61	21.10	78.3	36
171	Title	440	1	68	51.50	1.71	17.72	76.7	41
172	Title	340	1	68	42.40	1.59	16.88	80.7	32
173	Title	290	1	68	66.90	1.62	25.65	82.5	22
174	Title	450	1	68	63.20	1.60	24.69	80.2	59
175	Title	350	0	69	42.10	1.55	17.59	65.6	33
176	Title	460	0	69	56.90	1.59	22.65	82.4	73
177	Title	590	0	69	69.30	1.52	30.19	70.4	52
178	Title	470	0	69	49.90	1.49	22.51	73.1	52
179	Title	580	1	69	54.50	1.67	19.61		51
180	Title	430	1	69	69.30	1.71	23.62	75.3	54
181	Title	280	0	70	49.60	1.49	22.28	79.6	51
182	Title	450	0	70	56.40	1.51	24.83	73.6	49
183	Title	450	0	70	52.50	1.55	21.97	76.7	62
184	Title	450	1	70	66.40	1.69	23.17	55.2	47
185	Title	410	1	70	61.80	1.64	22.87	79.7	68
186	Title	360	0	71	52.00	1.51	22.70	73.5	41
187	Title	540	0	71	49.60	1.50	22.20	84.0	70
188	Title	440	0	71	47.21	0.02	21.00		64
189	Title	650	0	71	43.30	1.49	19.50	83.8	63
190	Title	430	0	72	56.30	1.62	21.70	94.4	42
191	Title	350	1	72	44.00	1.59	17.30	66.4	33
192	Title	350	0	73	54.20	1.62	20.78	82.0	36
193	Title	270	0	73	51.10	1.59	20.11	70.0	49
194	Title	350	0	73	69.40	1.53	29.60	67.0	22
195	Title	350	0	73	62.30	1.53	27.00	76.0	62
196	Title	360	1	74	53.80	1.58	21.70	85.0	22
197	Title	440	0	76	45.20	1.57	18.30	73.1	56
198	Title	200	1	78	73.70	1.63	27.60	83.3	26
199	Title	350	1	78	60.80	1.65	22.30		25

	Group H	Group I	Group J	Group K	Group L	Group M
	HR max	%HRmax	Distance 1	Distance 2	D2-D1	Total distance
	Y	Y	Y	Y	Y	Y
1	199	89	790	1020	230	1020
2	198		760	760	0	760
3	198		670	680	10	680
4	198	97	760	860	100	860
5	198	93	860	860	0	860
6	198	79	780	900	120	900
7	198	81	760	780	20	780
8	198	100	770	770	0	770
9	198	90	640	580	-60	640
10	198	91	670	780	110	780
11	198	90	550	560	10	560
12	198	54	780	670	-110	780
13	198	89	650	680	30	680
14	198	51	800	660	-140	800
15	198	83	640	690	50	690
16	198	55	430	450	20	450
17	198	78	640	530	-110	640
18	198	90	450	450	0	450
19	198	62	660	630	-30	660
20	198	91	650	770	120	770
21	198	88	850	960	110	960
22	198	90	690	791	101	790
23	198	88	640	660	20	660
24	198	80	610	670	60	670
25	198	72	650	670	20	670
26	198	79	910	940	30	940
27	198	91	860	880	20	880
28	198	87	750	970	220	970
29	197		610	650	40	650
30	197		660	660	0	660
31	197		690	670	-20	690
32	197		640	660	20	660
33	197		580	620	40	620
34	197	73	720	800	80	800
35	197	63	430	430	0	430
36	197	104	670	770	100	770
37	197	65	650	680	30	680
38	197	81	800	920	120	920
39	197	83	710	910	200	910
40	197	65	610	640	30	640
41	197	78	660	660	0	660
42	197		710	720	10	720

	Group H	Group I	Group J	Group K	Group L	Group M
	HR max	%HRmax	Distance 1	Distance 2	D2-D1	Total distance
	Y	Y	Y	Y	Y	Y
43	197		690	770	80	770
44	197		1020	1000	-20	1020
45	197		670	670	0	670
46	197		590	650	60	650
47	197	86	900	1020	120	1020
48	197	79	930	1020	90	1020
49	197	93	760	960	200	960
50	197	66	650	710	60	710
51	197	95	780	840	60	840
52	197	79	770	660	-110	770
53	197	61	920	800	-120	920
54	197	84	910	1020	110	1020
55	197	53	800	850	50	850
56	197	56	1020	940	-80	1020
57	196	86	700	790	90	790
58	196	94	570	750	180	750
59	196	60	350	640	290	640
60	196	68	650	770	120	770
61	196		560	580	20	580
62	196		660	640	-20	660
63	196		710	730	20	730
64	196		660	780	120	780
65	196		1020	1020	0	1020
66	196	80	1020	930	-90	1020
67	196	82	780	890	110	890
68	196	75	960	1020	60	1020
69	196	80	770	730	-40	770
70	196	77	1020	920	-100	1020
71	196	69	660	760	100	760
72	196	71	650	810	160	810
73	196	97	1020	1020	0	1020
74	196	62	910	890	-20	910
75	196	92	1020	1020	0	1020
76	195	67	440	390	-50	440
77	195	46	690	650	-40	690
78	195	75	430	460	30	460
79	195		770	780	10	780
80	195		1020	1020	0	1020
81	195		910	910	0	910
82	195		780	770	-10	780
83	195	90	920	890	-30	920
84	195	69	690	750	60	750

	Group H	Group I	Group J	Group K	Group L	Group M
	HR max	%HRmax	Distance 1	Distance 2	D2-D1	Total distance
	Y	Y	Y	Y	Y	Y
85	195	75	640	790	150	790
86	195	88	1020	1020	0	1020
87	195	89	1020	1020	0	1020
88	195	82	930	1020	90	1020
89	195	95	980	690	-290	980
90	195	73	920	890	-30	920
91	194	64	660	660	0	660
92	194	41	670	670	0	670
93	194		950	1020	70	1020
94	194		1020	1020	0	1020
95	194	79	650	780	130	780
96	194	90	990	930	-60	990
97	194	62	540	640	100	640
98	193		530	580	50	580
99	193		680	700	20	700
100	193	61	340	360	20	360
101	193	80	1020	1020	0	1020
102	193		570	530	-40	570
103	193	77	910	1020	110	1020
104	193	72	890	1020	130	1020
105	192		610	540	-70	610
106	191	90	810	1020	210	1020
107	190	84	770	910	140	910
108	190	95	900	1020	120	1020
109	189		1020	970	-50	1020
110	189	75	800	910	110	910
111	189	88	790	910	120	910
112	189		890	890	0	890
113	188	93	810	740	-70	810
114	188	102	680	420	-260	680
115	188		1020	1020	0	1020
116	188	99	1020	950	-70	1020
117	187	98	570	670	100	670
118	187		790	760	-30	790
119	186	78	570	660	90	660
120	185	63	370	320	-50	370
121	184	85	440	560	120	560
122	181	94	710	590	-120	710
123	180	87	270	440	170	440
124	179	69	650	550	-100	650
125	178		640	680	40	680
126	178	97	1020	540	-480	1020

	Group H	Group I	Group J	Group K	Group L	Group M
	HR max	%HRmax	Distance 1	Distance 2	D2-D1	Total distance
	Y	Y	Y	Y	Y	Y
127	176	87	440	440	0	440
128	174	91	340	340	0	340
129	173	83	340	430	90	430
130	172	91	640	550	-90	640
131	172	80	540	580	40	580
132	172	80	430	350	-80	430
133	167	63	370	350	-20	370
134	167	93	770	660	-110	770
135	166	104	450	530	80	530
136	166	57	470	450	-20	470
137	165	75	340	440	100	440
138	165	75	440	340	-100	440
139	165	69	460	440	-20	460
140	165	88	530	460	-70	530
141	164	71	220	270	50	270
142	164	47	260	200	-60	260
143	163	73	450	460	10	460
144	163	87	640	760	120	760
145	162	89	610	450	-160	610
146	161	101	580	650	70	650
147	161	71	370	490	120	490
148	161	91	550	760	210	760
149	160	79	460	540	80	540
150	159	93	290	350	60	350
151	159	94	450	450	0	450
152	159	86	280	430	150	430
153	159	89	560	580	20	580
154	158	77	350	430	80	430
155	158	54	370	350	-20	370
156	157	89	570	590	20	590
157	157	79	340	440	100	440
158	157	110	190	280	90	250
159	156	84	380	430	50	430
160	156	88	740	520	-220	740
161	155	91	390	400	10	400
162	155	86	380	440	60	440
163	155	79	270	280	10	280
164	154	88	370	360	-10	370
165	154	81	370	360	-10	370
166	154	71	200	210	10	210
167	154	71	350	340	-10	350
168	154	75	440	400	-40	440

	Group H	Group I	Group J	Group K	Group L	Group M
	HR max	%HRmax	Distance 1	Distance 2	D2-D1	Total distance
	Y	Y	Y	Y	Y	Y
169	154	82	350	350	0	350
170	152	83	440	360	-80	440
171	152	81	440	380	-60	440
172	152	78	340	270	-70	340
173	152	75	290	280	-10	290
174	152	91	440	450	10	450
175	151	70	350	340	-10	350
176	151	94	450	460	10	460
177	151	91	530	590	60	590
178	151	90	330	470	140	470
179	151	85	560	580	20	580
180	151	93	350	430	80	430
181	150	80	280	280	0	280
182	150	79	450	340	-110	450
183	150	103	320	450	130	450
184	150	77	360	450	90	450
185	150	88	220	410	190	410
186	149	97	360	360	0	360
187	149	94	450	540	90	540
188	149	91	440	440	0	440
189	149	99	620	650	30	650
190	148	99	370	430	60	430
191	148	62	350	280	-70	350
192	147	84	280	350	70	350
193	147	67	260	270	10	270
194	147	66	340	350	10	350
195	147	102	350	340	-10	350
196	146	59	300	360	60	360
197	144	99	360	440	80	440
198	142	76	200	140	-60	200
199	142	66	350	350	0	350
